# Treat-to-target approach in managing modifiable risk factors of patients with coronary heart disease in primary care in Singapore: what are the issues?

**DOI:** 10.1186/1447-056X-10-12

**Published:** 2011-09-22

**Authors:** Ngiap Chuan Tan, Sally Chih Wei Ho

**Affiliations:** 1 SingHealth Polyclinics-Pasir Ris, 1, Pasir Ris Drive 4, #01-11, 519457, Singapore; 2 DUKE-NUS Graduate Medical School Singapore, 8, College Road, 169857, Singapore; 3 NUS Yong Loo Lin School of Medicine, 21, Lower Kent Ridge Road, 119077, Singapore; 4 Department of Education and Research, Singhealth Polyclinics, 167, Jalan Bukit Merah, #15-10, Connection One (Tower 5), 150167, Singapore

**Keywords:** Coronary Heart Disease, primary care, risk factor

## Abstract

**Background:**

The key management strategy for established coronary heart disease (CHD) patients is to control the underlying risk factors. Further complications will be reduced when these risk factors are treated-to-target (TTT) as recommended by clinical practice guidelines. These targets include blood pressure (BP) lower than 130/80 mm Hg and LDL-cholesterol of less than 2.6 mmol/L and for those with type 2 diabetes mellitus (DM), HBA1c less than 7%. This article aimed to explore the issues affecting this approach from both the patients' and primary care physicians' (PCP) perspectives.

**Methods:**

The study involved triangulation of research methods to determine the findings. Part A: focus group discussions to collect qualitative data from patients with CHD and from PCPs who were managing them in primary care. Part B: A subsequent questionnaire survey to determine the extent of their awareness of treatment targets for modifiable risk factors.

**Results:**

CHD patients had variable awareness of the modifiable risk factors for CHD due to poor concordance between the PCPs' approach in managing the CHD patients and the latter's reception of information. 46% of participants knew their targets of BP control correctly; 11% of them were correct in stating their target for LDL-cholesterol control. Amongst these participants with DM (n = 146), 27% of them were correct in indicating their target of diabetic control.

**Conclusions:**

Communication and practice barriers exist which hinder the treat-to-target approach in mitigating the risk factors for CHD patients. Incorporating this approach in routine clinical practice by PCPs has greater potential to achieve treatment targets for patients.

## Background

Hypertension, dyslipidaemia and diabetes mellitus are prime risk factors for coronary heart disease (CHD). Global clinical practice guidelines recommend controlling these risk factors as a management strategy to mitigate the risk of recurrent CHD. Hence, physicians managing these patients should focus on treating these modifiable risk factors to evidence-based targets. However, most CHD patients failed to reach these targets [1,2]. In Singapore, deaths from CHD has only declined marginally from 19.8% in 2007 to 19.2% in 2009 [3] Moreover, Ho KT et al [4] reported that 70% of CHD patients in a cohort study of CHD patients from the Singapore National Cardiac Registry did not achieve a serum LDL-C target of < 100 mg/dL (2.6 mmol/L). 94% of the very high risk patients did not achieve the stringent serum LDL-C target of < 70 mg/dL (1.8 mmol/L).

Locally, patients with CHD are often discharged to primary care from cardiologists in tertiary institutions, once their conditions are stabilised after the acute cardiac events. Due to the dual healthcare system in Singapore, CHD patients can select their primary care physicians (PCP) based on their personal preference, either at the public polyclinics or the private general practitioner clinics. To reduce their CHD associated mortality and morbidity risks, more effective disease management in primary care is urgently needed. Secondary prevention of recurrent CHD by adopting "Treat-to-target" (TTT) approach towards their risk factors is being advocated a cluster of polyclinics in Singapore.

This study aimed to determine the issues associated with this "TTT" approach in managing the health of CHD patients in primary care in Singapore.

## Methods

A mixed-method was used in this study. A qualitative study (CAD study) which aimed to explore the management issues of PCP and their CHD patients was first carried out. This was followed by a cross-sectional quantitative study (HEALTH study) of a larger sampling of CHD patients, whose aim was to substantiate and complement the earlier qualitative data based on the same source population. This paper presents the results of this triangulation method from both a qualitative study and quantitative survey. Both studies were approved by SingHealth Polyclinics Institution Review Board.

### Part 1: CAD (Coronary Artery Disease) study

Focus group discussions (FGD), executed between September 2005 and March 2007, were used to gather qualitative data from (a) patients and separately, from (b) PCPs [5,6]. These target participants were identified by the investigators based on a case-encounter basis at the research sites. Snowball sampling method was also used to recruit potential participants. The latter were invited to take part in the FGD if they were able to understand and communicate in English. They were also screened for eligibility using the following criteria:

(a) The participants were adult CHD patients treated with the following modalities: percutaneous, transluminal coronary angioplasty (PTCA), coronary arterial bypass grafting (CABG) or non-invasive pharmacological treatment. They were diagnosed with CHD for at least one year, based on polyclinic medical records and confirmation based on referral documents from cardiologists.

(b) The PCPs included polyclinic doctors and general practitioners from singleton and group practices, who were managing CHD patients in the community.

Purposive selection of these participants from a variety of demographic profiles was conducted to ensure multivariate construct of the study population in both groups.

Investigators took turns to facilitate the FGDs based on semi-structured topic guide developed after mutual deliberations. All FGDs were audio-taped for subsequent transcription by independent transcribers. The investigators carried out debrief after each FGD. Any new concepts and ideas were noted and included in the topic guide for discussion in the subsequent FGD. The study was terminated after saturation of ideas as assessed by the investigators.

The investigators used the software package NVivo7 (QSR International Pty Ltd, Australia) to code the verbatim transcripts and organised them into emergent themes.

Based on findings generated by qualitative content analysis of the CAD study, themes considered to be important were included in the design of the questionnaire to be used in part two of the study.

### Part 2: HEALTH (Heart patients' Expectation of care, Awareness of disease, Lifestyle modifications, Targets of treatment and Health-seeking behaviour) study

This cross-sectional survey was a collaborative study between SingHealth Polyclinics and Ngee Ann Polytechnic School of Nursing. The investigators deliberated and designed the HEALTH study questionnaire based on preliminary qualitative data from the CAD study. The surveys were carried out by the polytechnic student nurses in the nine SingHealth Polyclinics from June 07 to September 07. These interviewers received briefings from the investigators to clarify implementation issues and to standardise the execution of the survey. They were supervised by their polytechnic tutors.

The participants satisfied the same inclusion and exclusion criteria of the CAD study. The questionnaire comprised thirty questions pertaining to CHD patients' expectation of care, awareness of disease, lifestyle modifications, targets of treatment and their health-seeking behaviours. The questionnaire content was derived from issues raised during the CAD study to ensure internal validity. No external validation was done due to absence of local precedent study and the simple design of the questions.

CHD participants were asked by the student nurses if they were aware of their treatment targets for blood pressure, LDL-C (LDL-cholesterol) and glycated haemoglobin (where relevant), with "yes", "no" and "don't know" answers. For affirmative answers, the participants were expected to identify correctly the appropriate target range based on multiple choices. Blood pressure is targeted at lower than130 mm Hg (systolic) and lower than 80 mm Hg (diastolic), which is standardised for all CHD patients based on the investigators' institution clinical practice guidelines.

Categorical variables were tabulated and analysed using Stata-10 software (StataCorp LP, USA). All investigators deliberated the results in both segments of the study. SingHealth Polyclinics institution review board approved the two studies.

## Results

**Figure 1:**
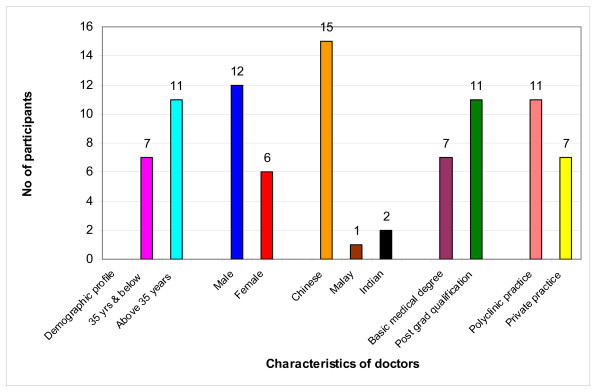
**Profile of doctors who participated in CAD Study**.

The demographic profiles of 44 participants (including 3 participants on follow up by private primary care clinics and 2 were managed at specialist clinics) in the CAD study are shown in Table 1 and that of 303 participants, of the HEALTH study are depicted in Table 2. The profiles of the 18 PCPs from 3 FGDs are shown in Figure 1.

**Table 1:** Demographic profile of participants in CAD Study

FGD* (n)	Gender		Age (years)		Race				Highest educational Level attained		Site of follow-up	
	Male	Female	≤ 60	> 60	Chinese	Malay	Indian	Others	Secondary or below	JC/Diploma/Tertiary	Polyclinic	GP/Specialist
*1 *(8)	6	2	5	3	7	0	1	0	6	2	8	0
*2 *(6)	5	1	2	4	3	1	1	1	4	2	6	0
*3 *(7)	6	1	4	3	4	1	2	0	7	0	6	1
*4 *(11)	10	1	5	6	7	0	2	2	8	3	8	3
*5 *(12)	10	2	7	5	7	1	3	1	10	2	11	1
Total (44)	37	7	23	21	28	3	9	4	35	9	39	5

*FGD: Focus Group Discussion (n: number of participants in the respective FGD)

**Table 2:** Demographic profile of 303 participants in HEALTH Study

Age (years)	No of patients (%)
41-60	79 (26.1)
61-70	87 (28.7)
71-80	137 (45.2)
Gender	
Male	182 (60.1)
Female	121 (39.9)
Ethnic groups	
Chinese	223 (73.6)
Malay	43 (14.2)
Indian	31 (10.2)
Others	6 (2.0)
Education	
Primary/Secondary	275 (90.8)
Junior college/diploma/tertiary	28 (9.2)
Duration of coronary heart disease (years)	
< 1	16 (5.3)
1-5	101 (33.3)
> 5	186 (61.4)

### Theme 1: Gap in communication between PCPs and CHD patients on specific goals of treatment, with differences in foci and expectation between the two parties during the consultation

CHD participants were generally satisfied when their PCPs reported that their disease control was adequate. They did not expect to be told of their specific treatment targets.

When asked if the doctors discussed the treatment target of blood pressure (BP) control with patients, polyclinic participant (aged 67 years, FGD3) replied: *"No. My doctor, that I last saw said: "Ok, your blood pressure is good", that's all. I don't know what is good, what is not good."*

Similarly in FGD4, when asked about treatment target for cholesterol, polyclinic participant A: *"I never ask the doctor how much. Never say good or no good." *Polyclinic participant B in the same FGD: *"They (doctors) say all my cholesterol is good."*

On the other hand, PCPs were preoccupied with clinical assessment of cardiac status to exclude any acute myocardial event in their consultation with CHD patients. Specifying treatment targets with their patients was not mentioned in all three FGDs. There was lack of discussion of mutually agreed goals of treatment between PCPs and their CHD patients.

### PCP (FGD1)

"I usually ask them whether they have any new symptoms, like chest pain, breathlessness on walking. If they say, well I'm, that's okay. So the next thing I would find out is whether they are compliant with their medications. If they're a bit on the obese side, I ask them if they are doing anything about diet control, and estimate their understanding of their weight, and some risk factors. If they're smoking, check whether they would stop. If all are okay, I would check their blood pressure. If the blood pressure is quite under control, then I would ask them if they are worried about anything else. Are they satisfied? Yes, everything is fine; I would just repeat the same medication. But if for example, the panel test is not done within the last year, I would advise that they do another one, and from the panel test, I can follow up next visit and see whether the cholesterol, and sugar etcetera are under control, or it's getting out of control."

In this study, it appeared that acute presentation of chest pain was a more common reason of consultation with PCPs in private clinics. In the context of potential litigation risk, their focus was to exclude potentially life-threatening acute cardiac event. PCPs were aware of the common risk factors of CHD but they did not generally communicate the specific treatment targets of these risk factors to their patients

### When asked about what took place in a consultation with CHD patients, PCP (FGD1)

"Management of ischemic heart disease is primarily history taking (and) risk assessment. In our practice, a lot of patients complain of chest pain. If we end up referring all of them to cardiologist, we are not exercising enough clinical judgement. We are liable... if the history is typical of chest pain, I will refer immediately to A and E (Accident and Emergency). But if the history and the rest of risk factors are not there, then I will do an ECG and do my own assessment. But there's still a small chance, but your risk is low. So I always discuss options with the patient. Sometimes the atypical chest pain may still be angina, or acute myocardial infarct. If I find that this patient is very demanding, I may jeopardize my license, so I will formally refer. In one month there are so many cases with chest pain you cannot afford to refer all of them. For those chronic cases, you realize that they are just musculoskeletal pain, you can just give painkiller."

### PCP (FGD1)

"In the private sector, we see two types of ischemic heart disease patients. Those fresh cases, they've never been worked up, they step into the consultation room and in the course of the consultation we realize that they may have underlying ischemic heart disease. The second group is the stable angina patient. They have been worked up somewhere else and decide to follow up in the clinic. For the new cases, if they come in with fairly new onset of angina symptoms, I think they deserve a more thorough cardiologist's review. By the time these patients come to us, they have other risk factors to take into consideration, like smoking, cholesterol. So looking at these risk factors, they would have some degree of narrowing of the coronary arteries. How severe (are the arteries affected), it is beyond our assessment at the primary care level. In a sense, we are still controlling symptoms. Chronic cases would be treated like those in the polyclinic."

### Theme 2: Difficulty in remembering the numerical treatment targets amongst CHD patients

### Patient (FGD4)

"I can't remember but I think LDL should be kept to within 60 (mg/dl) or what, I can't remember the exact figure."

CHD patients appeared to experience difficulty in remembering the treatment targets, with some incorrectly perceived it to be a variable target.

When asked about BP treatment target, Patient A (FGD2): *"120 (mmHg). More than 120... still on the high side." *In the same FGD, Patient B indicated *"130-140 (mmHg)" *as the target BP control and polyclinic participant 3 (aged 55 years): *"Fluctuate lah. This kind of thing (treatment target) fluctuates."*

**Figure 2:**
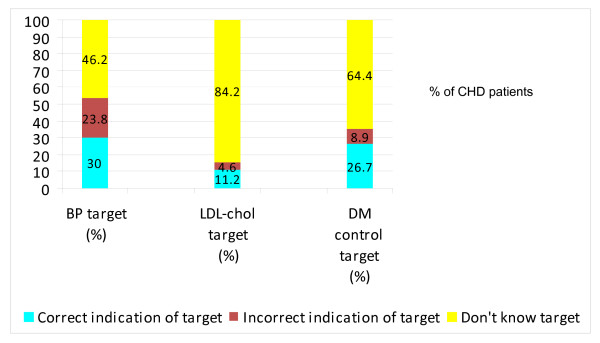
**Extent of awareness of treatment targets for BP, LDL-cholesterol and HBA1c (for Diabetes mellitus) amongst CHD patients (HEALTH study)**.

The lack of awareness of treatment targets is expounded In the HEALTH study. 30% of CHD patients were aware of the correct target BP control, 27% for correct HBA1c target level and 11% for the appropriate LDL-cholesterol level (Figure 2).

### Theme 3: Inadequate understanding of the glycated haemoglobin as the treatment target for CHD patients with diabetes mellitus

In this study, 48% of the CHD patients in the HEALTH study have type 2 diabetes mellitus. Amongst these diabetic CHD patients, there was confusion between serum glucose level and glycated haemoglobin as the treatment target. Awareness of the treatment target for diabetes mellitus was also lacking amongst these patients.

### Patient (FGD3)

*"For diabetes, the level should be six and below." *When probed by the moderator on the reference blood test, the same patient replied: *"It's the one that they poke (with) the needle. I can't remember the name of the test."*

### Patient (FGD2)

"What I understand (HBA1c), it is the three months' average. With the blood (glucose) test, you can cheat: you don't eat, your reading will be low. But with the three months' average, whether you eat or don't eat, it will show. I think it should be below ten; seven, eight, I think it's acceptable."

## Discussion

Effective prevention of recurrent CHD encompasses a comprehensive range of therapeutic interventions to manage the various risk factors, from lifestyle modifications, weight management, smoking cessation to medications [7]. This presents a challenge to the PCPs, who face time and resource constraints in their practices and the imperatives of other immediate issues raised by the CHD patients during the consultation. However, PCPs have the unique advantage of managing these patients from a holistic perspective with opportunities to recognise the various risk factors and commence preventive interventions. This entails a paradigm shift from merely counselling and motivating patients to embark on healthy lifestyles to empowering the patients to recognise their respective treatment targets. Discussion should focus on addressing the issues pertaining to lifestyle modifications [8] and negotiation on mutually agreed measures to achieve the treatment targets.

Whilst patient-dependent lifestyle modifications are important, PCPs should mitigate their patients' modifiable risk factors such as blood pressure, LDL-cholesterol and diabetic control by optimising physician-initiated pharmaceutical interventions to reach evidence-based treatment targets. Physician-centred intervention alone may be inadequate. To achieve these targets, they should communicate such goals to patients and negotiate for mutual agreement during the physician-patient consultation. The rationale and benefits behind this treat-to-target approach should be explained to patients in lay language, such that the latter can understand and embrace as an essential component of their CHD management.

45% of CHD patients in the HEALTH study were above 70 years of age and 98% of them had, at most, secondary education. In view of age and educational status, educating these patients to remember their treatment targets would require innovative measures. These include provision of aide-memoires, diaries and educational literature detailing target values to patients and their caregivers, developing enhanced health education, outreach programmes, quiz, games or other support group activities that focus on treatment targets. However such multi-modal interventions need further research evaluation to ascertain their cost-effectiveness.

Only 27% of diabetic CHD patients correctly identified the glycated haemoglobin target for their diabetic control. Whilst it is uncertain if understanding the true nature of the test makes any impact in their diabetic control, simplifying the diabetic treatment target to a single value may be easier for these patients to assimilate the information.

In the local setting, the private primary care clinics are serviced by off-site commercial laboratory vendors. In contrast, in-house laboratory service is provided within the public polyclinics. These polyclinics are affiliated to the respective regional public hospitals, which support their laboratory services. Laboratory reports for LDL-C in private clinics (in mg/L) and the public polyclinics (in mmol/L) differ in terms of units of the assay. This may further increase the difficulty of remembering the cholesterol treatment target amongst the patients. Whilst conversion tables are available, a standardised unit of measurement for LDL-C will facilitate the delivery of CHD-related information to patients with cardiovascular risk across the whole nation. Harmonisation of the laboratory reports is a potential solution and this will be implemented when the national electronic health record system rolls out in the near future.

While this study employed triangulation approach towards combining both qualitative and quantitative data, the subjects recruited were mainly CHD patients managed in public polyclinics and caution should be exercised in generalising the results to all CHD patients in Singapore. Most participants, who were recruited in the qualitative CAD study due to transcription constraints, spoke English during the FGD, which constitutes another limitation. The investigators did not include the data pertaining to whether the CHD patients achieved their treatment targets as such data are published in the official websites of the two clusters of polyclinics in Singapore.

## Conclusion

Treat-to-target approach in managing risk factors for CHD is hampered by a gap of communication between PCPs and patients on treatment targets, leading to low level of awareness of the latter. This may result in their failure to appreciate the relevance of achieving treatment target in managing their chronic condition.

## Competing interests

The authors declare that they have no competing interests.

## Authors' contributions

TNC designed and conceptualised the study, executed the study, analysed the qualitative and survey data and drafted the manuscript. SH facilitated and executed the FGD. All authors read and approved the final manuscript.
